# Complex Formation between Cytochrome *c* and a Tetra-alanino-calix[4]arene

**DOI:** 10.3390/ijms232315391

**Published:** 2022-12-06

**Authors:** Stefano Volpi, Aishling Doolan, Laura Baldini, Alessandro Casnati, Peter B. Crowley, Francesco Sansone

**Affiliations:** 1 Dipartimento di Scienze Chimiche, della Vita e della Sostenibilità Ambientale, Università degli Studi di Parma, Viale delle Scienze, 17/A, 43124 Parma, Italy; 2 School of Biological and Chemical Sciences, University of Galway, University Road, H91 TK33 Galway, Ireland

**Keywords:** chiral receptor, macrocycle, molecular recognition, protein

## Abstract

Owing to their remarkable features, calix[n]arenes are being exploited to study different aspects of molecular recognition, including protein complexation. Different complexation modes have been described, depending on the moieties that complement the aromatic cavity, allowing for function regulation and/or controlled assembly of the protein target. Here, a rigid cone calix[4]arene, bearing four anionic alanine units at the upper rim, was tested as a ligand for cytochrome *c*. Cocrystallization attempts were unfruitful, preventing a solid-state study of the system. Next, the complex was studied using NMR spectroscopy, which revealed the presence of two binding sites at lysine residues with dissociation constants (*K_d_*) in the millimolar range.

## 1. Introduction

The use of calix[n]arenes as ligands for proteins has been investigated with various aims, ranging from the discovery of biomedical tools [1,2,3,4] to the production of sensing devices [5,6] and advanced (soft) materials [7,8]. Different modes of interaction have been explored, taking advantage of the tunable size (*n* = 4, 5, 6, 8) and conformation of these macrocycles, and from the easy decoration of their aromatic cavity with a wide variety of functional groups. Tailored ligands directed at specific sites of functional proteins have been obtained via installation of peptide [9,10] and saccharide [11,12,13,14] moieties, making it possible to modulate the function of the selected molecular targets. On the other hand, less selective calixarenes bearing simple polar/charged functionalities can probe the surfaces of target proteins for appropriate binding sites. For example, cone para-guanidino-calix[4]arenes were found to plug the carboxylate-rich cavities of protein tetramers, enabling assembly and restoring or inhibiting biological activity [15,16]. Sulfonato- [17,18,19,20,21,22,23,24,25] or phosphonato-calix[n]arenes [25,26,27] have proven effective in the promotion of protein assembly and/or crystallization. These investigations revealed disparate binding modes, in which the macrocyclic ligand hosted amino acid side chains, and in some cases quasi-encapsulated the whole protein [17,18,22,27]. Moreover, calix[n]arenes allowed for the control of sophisticated processes such as dynamic protein assembly and porous framework fabrication [18,20,21,24].

**Figure 1:**
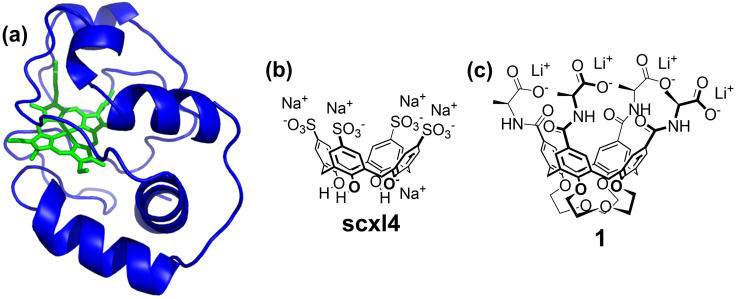
(**a**) Cartoon representation of cytochrome *c*, with the heme group and the protein backbone colored green and blue, respectively. (**b**) Tetrasulfonato-calix[4]arene **sclx_4_** that yielded the first cocrystal with this protein [18]. (**c**) Tetra-alanino-calix[4]arene-biscrown-3 **1** studied in this work.

Cytochrome *c*, a highly cationic redox protein (Figure 1a), is one of the most studied targets for calix[n]arene-based ligands, which, depending on the functionalization, bind to different regions of its surface. A remarkable example of solution-phase recognition was described in an early report, in which a calix[4]arene decorated with four anionic peptide loops inhibited the catalytic site of the protein by targeting the surrounding cationic protein surface [9]. Recently, extended frameworks were described in a series of cocrystals in which cytochrome *c* was complexed by different anionic calixarenes. The first structure to be resolved included the tetrasulfonato-calix[4]arene (**sclx_4_**, Figure 1b) that explored the protein surface for proper binding sites. The ligand simultaneously accommodated the alkyl part of lysine side chains in the aromatic cavity, while establishing salt bridges between the lysine ammonium groups and the sulfonate units [17]. The use of larger calix[n]arenes (*n* = 6, 8 [21,23,24,25,26]), with various functional units at their upper or lower rim (i.e., phosphonate [25,26,27], bromo [18], benzyl [23], phenyl [18], PEG [19], and carboxylate groups [22]), also resulted in cocrystallization, evidencing the great versatility of cytochrome *c* for this purpose.

Cytochrome *c* was used in this work to test the binding ability of a calix[4]arene, hereafter indicated as **1** (Figure 1c), bearing four alanine residues at the upper rim and two crown-3 ether motifs at the lower rim [28]. We envisaged that this compound could be a valuable ligand for the following reasons: (i) each alanine residue provides a negatively charged carboxylate that can complement the lysine-rich surface of cytochrome *c*; (ii) the crown ether chains impart a rigid cone conformation, which is a common motif in most cocrystals including calix[4]arenes [17,18,22,27]; (iii) previous studies evidenced its affinity for native amino acids, their methyl esters hydrochloride and ammonium cations [28] suggesting that the recognition of appropriate residues could be replicated on a protein; and (iv) it would be the first chiral calixarene to target a protein without being directed at a specific site of its surface. Here, we report the solution-state characterization of the complex between cytochrome *c* and **1**, as performed via NMR spectroscopy.

## 2. Results and Discussion

**Figure 2:**
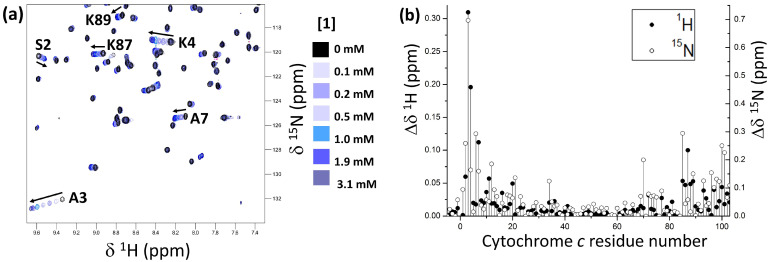
(**a**) Overlaid ^1^H–^15^N HSQC spectral region of 0.1 mM cytochrome *c* in the absence (black contours) or in the presence of 0.1–3.1 mM **1** (blue scale). (**b**) Chemical shift perturbation plots of cytochrome *c* amides at ∽30 eq. compound **1**. Cytochrome *c* residues are numbered from –5 to 103. Blanks correspond to proline residues 25, 30, 71, and 76, and unassigned G84.

Protein-ligand interactions were evaluated using ^1^H–^15^N HSQC NMR titrations at 30 °C in 20 mM potassium phosphate buffer, 50 mM NaCl, 1 mM sodium ascorbate, and 10% D_2_O, with pH 6.0 [17,27,29]. The amide resonances of ^15^N-enriched cytochrome *c* were monitored in the presence of increasing aliquots of **1** (Figure 2 and Figure S2). The amide NH signals experienced progressive chemical shift perturbations (Δδ) as a function of calixarene concentration, indicating fast exchange between the ligand-bound and -free states on the NMR timescale. No significant line broadening occurred in the tested concentration range, excluding the occurrence of aggregation processes. Two groups of resonances, assigned to residues around K4 and around K87/K89, were significantly affected by the addition of **1**, with A3 undergoing the highest chemical shift change (^1^H^N^ Δδ = 0.31 ppm).

**Figure 3:**
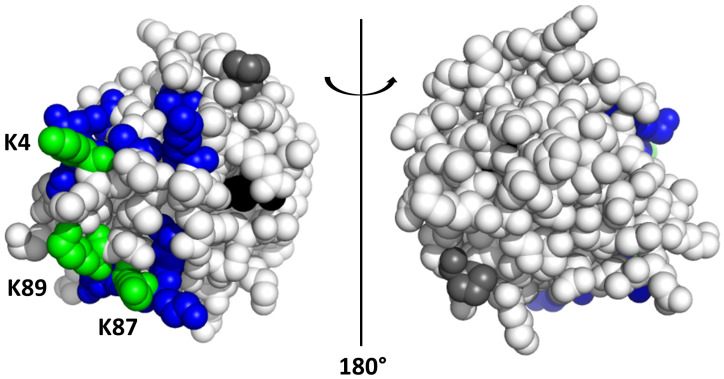
Binding map for compound **1** on cytochrome *c*. K4, K87, and K89 are colored green, and other residues with a significant chemical shift perturbation (Δδ ^1^H^N^≥ 0.04 or ^15^N^H^ ≥ 0.4 ppm) are blue. The heme and prolines are black and grey, respectively.

These observations are consistent with a preference for lysine binding, as demonstrated in the solid state for other anionic calix[4]arenes that yielded compatible NMR data [17,18,27]. For example, the solution-phase behavior of compound **1** is comparable with that of **sclx_4_**, which bound K4 and K89 in co-crystals with cytochrome *c* [17]. Thus, it is reasonable to consider these residues as good candidates for interaction with **1**, together with K87, which also experienced a large chemical shift perturbation. Mapping the highest chemical shift changes (Δδ ^1^H^N^ ≥ 0.04 ppm/^15^N^H^ ≥ 0.4 ppm) onto the surface of cytochrome *c* (Figure 3) revealed two small patches around these three lysines as the binding sites for compound **1**.

**Figure 4:**
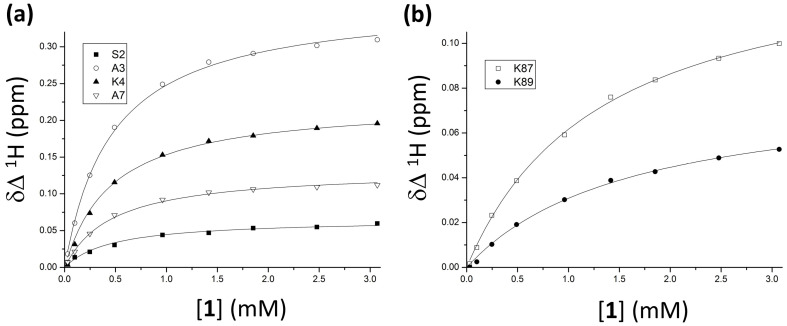
Experimental points and calculated binding curves for the two binding sites including (**a**) the group of residues around K4, and (**b**) K87 and K89. The experimental Δδ were fitted as a function of the concentration of **1** to a 1:1 binding model.

NMR data were also suitable for an estimation of the dissociation constants for the cytochrome *c*-**1** complex. A non-linear regression of the Δδ against the calix[4]arene concentration with a 1:1 binding model yielded *K_d_* values of circa 0.4 mM and 1.1 mM for the two clusters of residues flanking K4 and K87/K89, respectively (Figure 4, Section 3.2). The moderate affinity obtained by fitting the experimental data suggested a dynamic targeting of the protein by compound **1**, as observed for other calix[4]arene-based ligands [17,18,27]. ITC titrations [18] were attempted to collect additional thermodynamic information, but insufficient heat of complexation was obtained, consistent perhaps with the low affinity between the two binding partners (Section 3.3).

The present investigation allowed the identification of the main features of the cytochrome *c*-**1** interaction, but other aspects remain to be addressed to fully clarify the binding mode of **1** to this protein. However, with the caution that is due when comparing solution-state and solid-state data, the available crystal structures involving other calix[4]arenes help formulate some hypotheses. For example, **sclx_4_** [17] was reported to recognize the cytochrome *c* lysine residues with a mode of binding that was replicated by other calix[4]arene ligands (both sulfonated [18,22] and phosphonated [27]) approaching the same protein regions. Typically, the lysine ammonium group was complexed by the anionic calix[4]arene via charge–charge interactions, whereas the alkyl part of the side chain was encapsulated in the aromatic cavity forming CH-π non covalent bonds [17]. In principle, compound **1** could reproduce this mechanism using its four anionic carboxylates and its rigid, conical scaffold. Compared to the reference ligands with compact substituents, the alanine spacer may impart detrimental features, such as a longer distance between the anionic groups and the aromatic scaffold, a higher conformational mobility, and possibly steric hindrance. On the other hand, the presence of calixarene pseudopeptide groups (ArCONH) or terminal carboxylate groups could give rise to additional attractive interactions (H-bonds/electrostatic) with the adjacent protein surface. As an alternative, different modes of binding, such as salt bridges formation without inclusion of the rest of the side chain [22], exo-binding to the aromatic cavity [27], and ligand dimerization [18], were also observed in cocrystals, including the same protein and other lysine-binding calix[4]arenes, providing examples that would be equally compatible with the structure of **1**. Moreover, novel modes of binding might be favored by the presence of the chiral alanine substituents at the upper rim, which is a peculiar feature of this ligand.

Accordingly, different explanations could be provided regarding NMR data (Figure 2, Figure 3 and Figure 4), indicating that a crystal structure would be helpful to fully describe the protein-**1** interaction. Despite generally reported good agreement, significant differences between solution- and solid-state behaviors of a complex could be observed [17,18,27], increasing the risk of over-interpretation of the two types of data when systems including different ligands are compared. For the moment, compound **1** resisted cocrystallization with cytochrome *c* (Section 3.4). Cocrystallization trials yielded gels, liquid–liquid phase separation, or precipitates (Figure S3). The challenge of cocrystallization may be due to the higher flexibility of the alanine substituents with respect to the simpler phosphonate or sulfonate groups [17,18,22,27]. On the other hand, other calixarenes that initially proved challenging were subsequently found to yield cocrystals [20], suggesting that this issue could be resolved by further experiments.

## 3. Materials and Methods

### 3.1. General Information

All reagents were purchased from Merck and used as such. Compound **1** was synthesized according to a literature procedure [28] and purity was assessed via ^1^H NMR (Figure S1). Established protocols were used to obtain ^15^N-labeled and unlabeled Saccharomyces cerevisae cytochrome *c* C102T [30,31,32]. A Bruker AV500 spectrometer recorded ^1^H NMR spectra and δ values were expressed in ppm relative to D_2_O (4.79 ppm at 25 °C). A 600 MHz Varian spectrometer equipped with a HCN cold probe acquired ^1^H–^15^N HSQC spectra with spectral widths of 16 ppm (^1^H) and 40 ppm (^15^N). ITC investigations were performed using a standard volume Nano ITC system equipped with a Hastelloy cell (TA Instruments).

### 3.2. NMR Titrations

Typical samples of ^1^H–^15^N HSQC titrations contained 0.1 mM ^15^N-labeled cytochrome *c*, 20 mM potassium phosphate buffer, 50 mM NaCl, 1 mM sodium ascorbate, and 10% D_2_O at pH 6.0. The experiments were performed at 30 °C by adding 0.6–15 μL aliquots of a 25 mM stock aqueous solution of **1**. Over the course of the titrations, the protein was diluted by 1.14-fold and samples were corrected to pH 6.0 ± 0.05 for each addition of **1**. Ligand-induced chemical shift perturbations (CSPs, Δδ) were analyzed with respect to the spectrum of pure cytochrome *c* (Figure S2) [31,33]. Titrations were repeated three times to ensure reproducibility, and binding isotherms were obtained by plotting the magnitude of the chemical shift change (Δδ) as a function of the concentration of **1**. Data were fit to a 1:1 binding model with the “bindfit” tool www.supramolecular.org [34,35], according to the equations:

(1) $${\Delta \delta =\delta }_{\max}\left(\frac{\left[\mathrm{HG}\right]}{{\left[H\right]}_{0}}\right),\;\left[\mathrm{HG}\right]=\frac{1}{2}\left({\left[G\right]}_{0}+{\left[H\right]}_{0}+\frac{1}{K_{a}}\;\right)-\sqrt{{\left({\left[G\right]}_{0}+{\left[H\right]}_{0}+\frac{1}{K_{a}}\;\right)}^{2}+4{\left[H\right]}_{0}{\left[G\right]}_{0}}$$

where [H]_0_ and [G]_0_ are the total concentrations of cytochrome *c* and **1**, respectively, and the endpoint δ_max_ and the association constant *K_a_* are the fitting parameters.

### 3.3. ITC Titrations

Cytochrome *c* was oxidized using potassium ferricyanide [36]. Protein and ligand stock solutions were dialyzed overnight against 20 mM potassium phosphate buffer and 50 mM NaCl at pH 6.0, and their concentrations were checked via UV-visible spectroscopy (ε_550_ = 28.5 mM^−1^·cm^−1^, and ε_270_ = 78.1 mM^−1^·cm^−1^, respectively [30]) and adjusted to the target values. Titrations were performed at 30 °C after centrifugation (4500 rpm, 10 min) and degassing (586 mm Hg, 8 min) of the samples. In a typical experiment, 24 × 10 μL aliquots of **1** (2 mM) were added at 400 s intervals to a 50 μM solution of the protein. Ligand into buffer and buffer into protein control titrations were performed in the same way and yielded negligible heat of dilution. In the tested experimental conditions, it was not possible to detect significant heat of cytochrome *c*-**1** complexation. Attempts to generate detectable heats of complexation by means of more concentrated stock solutions failed because of the limited solubility of the ligand in the tested buffer.

### 3.4. Cocrystallization Tests

The hanging- and sitting-drop vapor diffusion methods [37] were used for crystallization tests of the cytochrome *c*-**1** complex at 20 °C. Samples for hanging-drop configurations were prepared in 24-well plates by mixing 1 μL volumes of the reduced protein, the ligand, and the reservoir solution. Control drops were obtained by replacing the solution of the ligand with 1 μL of water. The sitting-drop setup was used with an Oryx8 robot (Douglas Instruments) and a 96-component screen JCSG HTS++ (Jena Bioscience), with protein and ligand stocks at 1.7 mM and 10 or 20 mM, respectively. Gels resulted from 22 of the tested conditions, 11 of which contained phosphate citrate or sodium citrate buffer (Figure S3a). Control drops devoid of **1** remained clear. Manual hanging-drop optimizations of the process were attempted using: (a) 1.7 mM cytochrome *c*; (b) 1.7, 10, or 20 mM **1**; and (c) 50 mM sodium citrate or phosphate citrate buffer (pH 5 or 4.2, respectively) and 10–30% PEG 3350 or PEG 8000. Most mixtures resulted in gels (Figure S3b). Amorphous precipitates were obtained by lowering the protein concentration (0.1 and 0.5 mM), whereas clear drops were obtained at a higher buffer concentration or pH (100 mM or pH 6 for both buffers). Other manual hanging-drop investigations were performed by combining: (a) 1.7 mM cytochrome *c*; (b) 1.7 or 50 mM **1**; and (c) 50 mM sodium citrate (pH 5) and 25% PEG 8000 in the presence of Na_2_SO_4_, Li_2_SO_4_, NaCl, NH_4_OAc, or NaOAc as additives at 50 mM or 1 M concentration. However, no crystals were obtained.

## 4. Conclusions

This study demonstrates, for the first time, a chiral peptido-calix[4]arene (**1**) in complex with a protein (cytochrome *c*) without targeting a specific site [9,10]. A structural characterization and affinity determination was performed using ^1^H–^15^N HSQC NMR experiments, revealing some similarities with previously established ligands for this protein. Binding at two protein regions, including K4 and K87-K89, was consistent with the binding sites of sulfonated [17,18,22] or phosphonated [27] calix[4]arenes. Dissociation constants in the millimolar range were consistent with a weak, dynamic interaction at the cytochrome *c* surface, in agreement with previous reports for similar protein-ligands systems [17,18,27]. Further investigations are required to fully clarify the protein recognition mechanism and the ability of compound **1** to mediate cytochrome *c* assembly. Cocrystallization tests will be performed in the future, as they would facilitate study of a complex including a new type of calix[4]arene-based ligand in the solid state. The enantiomer of compound **1** will be also included in these studies, to assess whether the replacement of the L-alanine units at the upper rim with their D-stereoisomers could generate a differential interaction with the protein.

## Data Availability

Data are available from the authors upon request.

## Supplementary Materials

The following supporting information can be downloaded at: https://www.mdpi.com/article/10.3390/ijms232315391/s1.

## References

[1] Ma X., Zhao Y. (2015). Biomedical Applications of Supramolecular Systems Based on Host-Guest Interactions. Chem. Rev..

[2] Hof F. (2016). Host-Guest Chemistry That Directly Targets Lysine Methylation: Synthetic Host Molecules as Alternatives to Bio-Reagents. Chem. Commun..

[3] Baldini L., Casnati A., Sansone F. (2020). Multivalent and Multifunctional Calixarenes in Bionanotechnology. Eur. J. Org. Chem..

[4] Pan Y.C., Hu X.Y., Guo D.S. (2021). Biomedical Applications of Calixarenes: State of the Art and Perspectives. Angew. Chem. Int. Ed..

[5] Pinalli R., Pedrini A., Dalcanale E. (2018). Biochemical Sensing with Macrocyclic Receptors. Chem. Soc. Rev..

[6] Kumar R., Sharma A., Singh H., Suating P., Kim H.S., Sunwoo K., Shim I., Gibb B.C., Kim J.S. (2019). Revisiting Fluorescent Calixarenes: From Molecular Sensors to Smart Materials. Chem. Rev..

[7] Yu G., Jie K., Huang F. (2015). Supramolecular Amphiphiles Based on Host-Guest Molecular Recognition Motifs. Chem. Rev..

[8] Crowley P.B. (2022). Protein–Calixarene Complexation: From Recognition to Assembly. Acc. Chem. Res..

[9] Hamuro Y., Calama M.C., Park H.S., Hamilton A.D. (1997). A Calixarene with Four Peptide Loops: An Antibody Mimic for Recognition of Protein Surfaces. Angew. Chem. (Int. Ed. Engl.).

[10] Sebti S.M., Hamilton A.D. (2000). Design of Growth Factor Antagonists with Antiangiogenic and Antitumor Properties. Oncogene.

[11] Cecioni S., Lalor R., Blanchard B., Praly J.P., Imberty A., Matthews S.E., Vidal S. (2009). Achieving High Affinity towards a Bacterial Lectin through Multivalent Topological Isomers of Calix[4]arene Glycoconjugates. Chem.—Eur. J..

[12] André S., Sansone F., Kaltner H., Casnati A., Kopitz J., Gabius H.J., Ungaro R. (2008). Calix[n]Arene-Based Glycoclusters: Bioactivity of Thiourea- Linked Galactose/Lactose Moieties as Inhibitors of Binding of Medically Relevant Lectins to a Glycoprotein and Cell- Surface Glycoconjugates and Selectivity among Human Adhesion/Growth-Regulatory. ChemBioChem.

[13] André S., Grandjean C., Gautier F.M., Bernardi S., Sansone F., Gabius H.J., Ungaro R. (2011). Combining Carbohydrate Substitutions at Bioinspired Positions with Multivalent Presentation towards Optimising Lectin Inhibitors: Case Study with Calixarenes. Chem. Commun..

[14] Garcia-Hartjes J., Bernardi S., Weijers C.A.G.M., Wennekes T., Gilbert M., Sansone F., Casnati A., Zuilhof H. (2013). Picomolar Inhibition of Cholera Toxin by a Pentavalent Ganglioside GM1os-Calix[5]Arene. Org. Biomol. Chem..

[15] Martos V., Bell S.C., Santos E., Isacoff E.Y., Trauner D., De Mendoza J. (2009). Calix[4]arene-Based Conical-Shaped Ligands for Voltage-Dependent Potassium Channels. Proc. Natl. Acad. Sci. USA.

[16] Gordo S., Martos V., Santos E., Menéndez M., Bo C., Giralt E., De Mendoza J. (2008). Stability and Structural Recovery of the Tetramerization Domain of P53-R337H Mutant Induced by a Designed Templating Ligand. Proc. Natl. Acad. Sci. USA.

[17] McGovern R.E., Fernandes H., Khan A.R., Power N.P., Crowley P.B. (2012). Protein Camouflage in Cytochrome c–Calixarene Complexes. Nat. Chem..

[18] Doolan A.M., Rennie M.L., Crowley P.B. (2018). Protein Recognition by Functionalized Sulfonatocalix[4]arenes. Chem.—Eur. J..

[19] Mummidivarapu V.V.S., Rennie M.L., Doolan A.M., Crowley P.B. (2018). Noncovalent PEGylation via Sulfonatocalix[4]arene—A Crystallographic Proof. Bioconjug. Chem..

[20] Rennie M.L., Fox G.C., Pérez J., Crowley P.B. (2018). Auto-Regulated Protein Assembly on a Supramolecular Scaffold. Angew. Chem. Int. Ed..

[21] Engilberge S., Rennie M.L., Dumont E., Crowley P.B. (2019). Tuning Protein Frameworks via Auxiliary Supramolecular Interactions. ACS Nano.

[22] Alex J.M., Brancatelli G., Volpi S., Bonaccorso C., Casnati A., Geremia S., Crowley P.B. (2020). Probing the Determinants of Porosity in Protein Frameworks: Co-Crystals of Cytochrome c and an Octa-Anionic Calix[4]arene. Org. Biomol. Chem..

[23] Mockler N.M., Ramberg K.O., Guagnini F., Raston C.L., Crowley P.B. (2021). Noncovalent Protein-Pseudorotaxane Assembly Incorporating an Extended Arm Calix[8]Arene with α-Helical Recognition Properties. Cryst. Growth Des..

[24] Ramberg K.O., Engilberge S., Skorek T., Crowley P.B. (2021). Facile Fabrication of Protein-Macrocycle Frameworks. J. Am. Chem. Soc..

[25] Mockler N.M., Engilberge S., Rennie M.L., Raston C.L., Crowley P.B. (2021). Protein-Macrocycle Framework Engineering: Supramolecular Copolymerisation with Two Disparate Calixarenes. Supramol. Chem..

[26] Rennie M.L., Doolan A.M., Raston C.L., Crowley P.B. (2017). Protein Dimerization on a Phosphonated Calix[6]Arene Disc. Angew. Chem. Int. Ed..

[27] Alex J.M., Rennie M.L., Volpi S., Sansone F., Casnati A., Crowley P.B. (2018). Phosphonated Calixarene as a “Molecular Glue” for Protein Crystallization. Cryst. Growth Des..

[28] Sansone F., Barboso S., Casnati A., Sciotto D., Ungaro R. (1999). A New Chiral Rigid Cone Water Soluble Peptidocalix[4]arene and Its Inclusion Complexes with α-Amino Acids and Aromatic Ammonium Cations. Tetrahedron Lett..

[29] Fielding L. (2007). NMR Methods for the Determination of Protein-Ligand Dissociation Constants. Prog. Nucl. Magn. Reson. Spectrosc..

[30] Crowley P.B., Chow E., Papkovskaia T. (2011). Protein Interactions in the Escherichia Coli Cytosol: An Impediment to In-Cell NMR Spectroscopy. ChemBioChem.

[31] Volkov A.N., Vanwetswinkel S., de Water K., van Nuland N.A.J. (2012). Redox-Dependent Conformational Changes in Eukaryotic Cytochromes Revealed by Paramagnetic NMR Spectroscopy. J. Biomol. NMR.

[32] Studier W.F. (2005). Protein Production by Auto-Induction in High-Density Shaking Cultures. Protein Expr. Purif..

[33] Delaglio F., Grzesiek S., Vuister G.W., Zhu G., Pfeifer J., Bax A. (1995). NMRPipe: A Multidimensional Spectral Processing System Based on UNIX Pipes. J. Biomol. NMR.

[34] Thordarson P. (2011). Determining Association Constants from Titration Experiments in Supramolecular Chemistry. Chem. Soc. Rev..

[35] Hibbert D.B., Thordarson P. (2016). The Death of the Job Plot, Transparency, Open Science and Online Tools, Uncertainty Estimation Methods and Other Developments in Supramolecular Chemistry Data Analysis. Chem. Commun..

[36] Rennie M.L., Crowley P.B. (2019). A Thermodynamic Model of Auto-Regulated Protein Assembly by a Supramolecular Scaffold. ChemPhysChem.

[37] Krauss I.R., Merlino A., Vergara A., Sica F. (2013). An Overview of Biological Macromolecule Crystallization. Int. J. Mol. Sci..

