# Supplementary material for: Complex Formation between Cytochrome c and a Tetra-alanino-calix[4]arene
Source: Int J Mol Sci. 2022 Dec 6;23(23):15391. doi: 10.3390/ijms232315391 (PMC9737847; doi:10.3390/ijms232315391)
Supplement: Supplementary file 1 [file ijms-23-15391-s001.zip › ijms-2063914-supplementary.pdf]

Complex Formation between Cytochrome *c* and a Tetra-alanino-calix[4]arene

Stefano Volpi [1], Aishling Doolan [2], Laura Baldini [1,*], Alessandro Casnati [1], Peter B. Crowley [2] and Francesco Sansone [1]

[1] Dipartimento di Scienze Chimiche, della Vita e della Sostenibilità Ambientale, Università degli Studi di Parma, Viale delle Scienze, 17/A, 43124 Parma, Italy
[2] School of Biological and Chemical Sciences, University of Galway, University Road, H91 TK33 Galway, Ireland
* Correspondence: laura.baldini@unipr.it

Supporting Information

Table of contents

Figure S1: ^{1}H NMR spectrum (D_2O, 25°C, 500 MHz) of compound **1** (page S2).

Figure S2: Overlaid ^{1}H-^{15}N HSQC full-spectra of 0.1 mM cytochrome *c* in the absence or in the presence of 0.1–3.1 mM **1** (page S2).

Figure S3: Gel formation observed during crystallization tests for the cytochrome *c* - **1** complex (page S3).

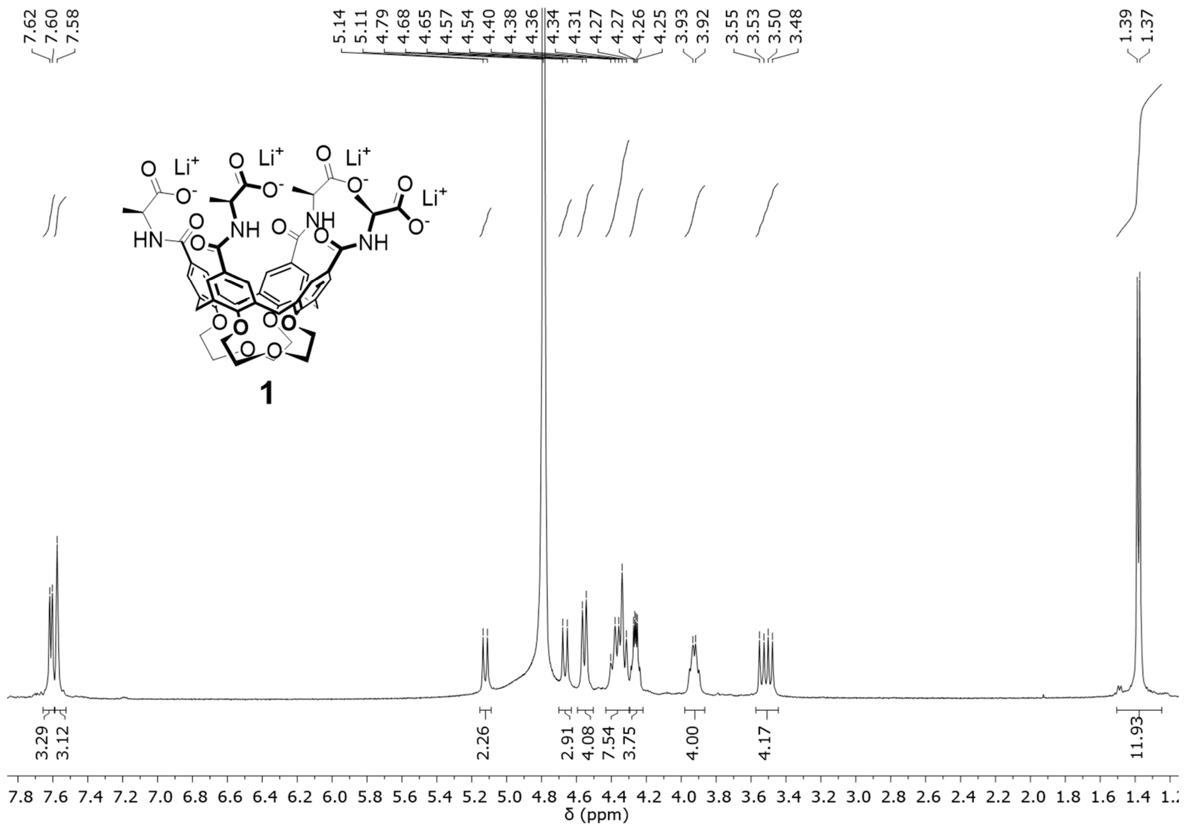

Figure S1: ^{1}H NMR spectrum (D_2O, 25°C, 500 MHz) of compound **1**.

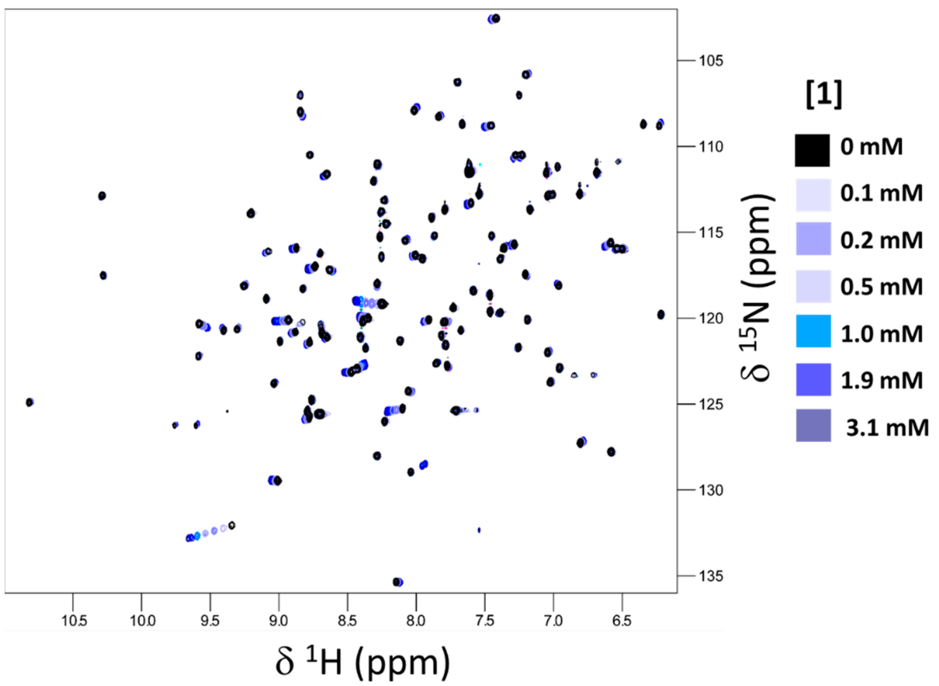

Figure S2: Overlaid ^{1}H-^{15}N HSQC full-spectra of 0.1 mM cytochrome *c* in the absence (black contours) or presence of 0.1–3.1 mM **1** (blue scale).

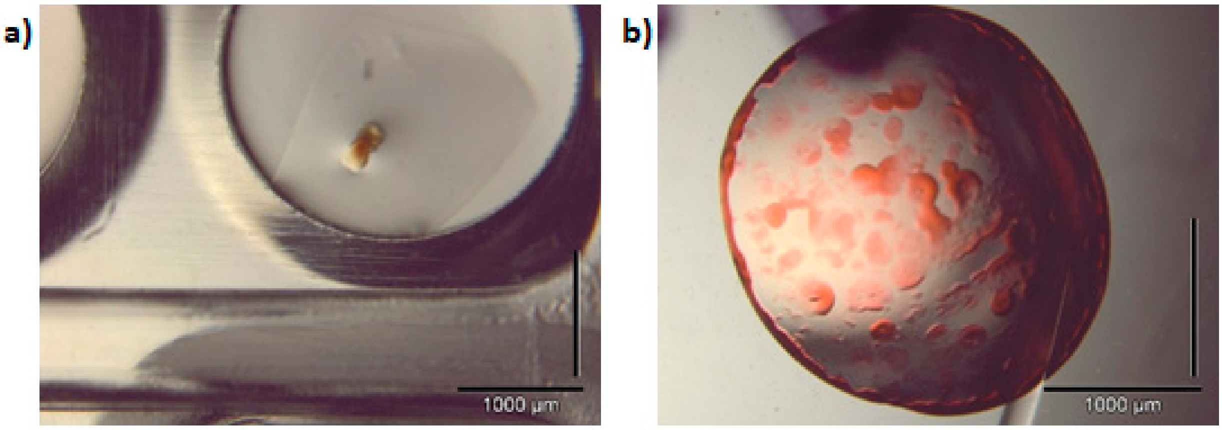

Figure S3: Gel formation observed during crystallization tests for the cytochrome *c* - **1** complex performed by **a)** a robot sitting drop and **b)** manual hanging drop vapor diffusion method (1.7 mM protein, 10 mM ligand). The reservoir solutions were **a)** 20% PEG 6000, 1M LiCl, 100mM sodium citrate buffer (pH 4) and **b)** 25% PEG 8000, 50mM sodium citrate buffer (pH 5).
